# Comparison of four ^11^C-labeled PET ligands to quantify translocator protein 18 kDa (TSPO) in human brain: (*R*)-PK11195, PBR28, DPA-713, and ER176—based on recent publications that measured specific-to-non-displaceable ratios

**DOI:** 10.1186/s13550-017-0334-8

**Published:** 2017-10-16

**Authors:** Masahiro Fujita, Masato Kobayashi, Masamichi Ikawa, Roger N. Gunn, Eugenii A. Rabiner, David R. Owen, Sami S. Zoghbi, Mohamad B. Haskali, Sanjay Telu, Victor W. Pike, Robert B. Innis

**Affiliations:** 1 Molecular Imaging Branch, National Institute of Mental Health, Bldg. 10, Rm. B1D43, 10 Center Drive, MSC-1026, Bethesda, MD 20892-1026 USA; 2 Imanova Ltd, London, UK; 3 Division of Brain Sciences, Department of Medicine, Imperial College, London, UK; 4 Centre for Neuroimaging Sciences, Institute of Psychiatry, Psychology and Neuroscience, King’s College, London, UK

**Keywords:** Neuroinflammation, Radiometabolites, Specific-to-non-displaceable ratio, Single nucleotide polymorphism, *BP*_ND_

## Abstract

Translocator protein (TSPO) is a biomarker for detecting neuroinflammation by PET. ^11^C-(*R*)-PK11195 has been used to image TSPO since the 1980s. Here, we compared the utility of four ^11^C-labeled ligands—(*R*)-PK11195, PBR28, DPA-713, and ER176—to quantify TSPO in healthy humans. For all of these ligands, *BP*_ND_ (specific-to-non-displaceable ratio of distribution volumes) was measured by partially blocking specific binding with XNBD173 administration. In high-affinity binders, DPA-713 showed the highest *BP*_ND_ of 7.3 followed by ER176 (4.2), PBR28 (1.2), and PK11195 (0.8). Only ER176 allows the inclusion of low-affinity binders because of little influence of radiometabolites and high *BP*_ND_. If inclusion of all three genotypes is important for study logistics, ER176 is the best of these four radioligands for studying neuroinflammation.

## Background

^11^C-(*R*)-PK11195 was introduced in the 1980s to image the inflammatory marker translocator protein 18 kDa (TSPO) in several brain disorders. Since then, many second-generation radioligands have been developed, but uncertainty exists as to whether these are any better than the prototypical agent ^11^C-(*R*)-PK11195. The uncertainty derives largely from two factors. First, until recently, receptor blocking studies have not been performed in human subjects, and such studies are the ‘gold standard’ method of measuring the percentage of specific uptake of a PET radioligand. Second, the sensitivity of ligand binding to genotype requires genotype testing for each study participant. This effect of genotype was first reported in relation to “non-binders” to ^11^C-PBR28, which may have the greatest genotype sensitivity of all TSPO PET radioligands. Owen and colleagues discovered that this non-binding was caused by the single nucleotide polymorphism (SNP) rs6971 [1]. This co-dominantly expressed SNP generates three genotypes: homozygous high-affinity binders (HABs), heterozygous mixed affinity binders (MABs), and homozygous low affinity binders (LABs). This discovery raised questions about the relative sensitivity of the prototypical and second-generation radioligands and about whether LABs provide adequate signal in brain to justify scanning them. For the four radioligands this commentary deals with, in vitro assays reported the following LAB/HAB ratios of inhibition constant, *K*i; PK11195: 0.79, PBR28: 55, DPA-713: 4.43 [2], and ER176: 1.28 [3].

The present commentary reviews data from recent studies conducted in our two laboratories (at the NIMH in the USA and at Imanova in the UK) that definitively show that three second-generation radioligands—^11^C-PBR28, DPA-713, and ER176—have much greater specific binding in human subjects than in ^11^C-(*R*)-PK11195. We also present the unexpected finding that ER176 appears unique in not generating radiometabolites that enter brain; as discussed below, this suggests that ER176 may be able to reliably quantify TSPO in LABs.

## Measuring the percentage specific binding in humans

Receptor blocking studies in animals found that some second-generation radioligands had much greater specific binding than ^11^C-(*R*)-PK11195. For example, the specific uptake of ^11^C-PBR28 in monkey brain is more than 10 times than that of ^11^C-(*R*)-PK11195 [4, 5]. Because species commonly differ regarding the density and affinity of the imaging target as well as radioligand metabolism, blocking studies must also be performed in humans to compare radioligands in particular for TSPO, which has about 20 times difference in receptor density between human and rhesus monkey [4, 6]. However, such blocking studies in humans are often impossible, either because the drug is not available for human use or because the necessary pharmacological dose of a given agent has unacceptable side effects. Fortunately, regarding TSPO, the blocking drug XBD173 is well-tolerated in humans. In a seminal translational paper, Rupprecht and colleagues reported that XBD173 decreased anxiety-like behaviors in rats and reduced the number and severity of panic attacks in humans [7]. XBD173 was studied as an anxiolytic agent because—like most TSPO ligands—it increases production of steroids and neurosteroids; the latter may have anti-anxiety properties.

For the four ^11^C-radioligands of interest ((*R*)-PK11195, PBR28, DPA-713, and ER176), our studies scanned healthy subjects at baseline and after an oral dose of XBD173 (10–90 mg). The baseline scan provided total distribution volume (*V*_T_), which is the sum of specifically bound (*V*_S_) and non-displaceable uptake (*V*_ND_). The baseline and blocked scans were analyzed with the Lassen/occupancy plot to provide *V*_ND_ [8]. This plot performs linear regression for baseline *V*_T_ (*x*-axis) and differences in *V*_T_ between baseline and blocked scans (*y*-axis) under the assumption that *V*_ND_ and the fraction of receptor occupancy are the same across brain regions with different receptor densities. Complete binding blockade is not required to perform the linear regression because what is required is the same fractional changes of specific binding across regions. *V*_ND_ obtained by Lassen/occupancy plot and *V*_T_ in the baseline scans allow calculation of the ratio of *V*_S_ (=*V*_T_ − *V*_ND_) to *V*_ND_, which equals *BP*_ND_, which can be roughly regarded as the ‘signal to noise ratio’ for a PET radioligand. In this context, *BP*_ND_ is a better measure than *V*_T_ because *BP*_ND_ quantifies the specific binding component directly, rather than just the sum of specific and non-specific binding (*V*_T_). Higher *BP*_ND_ rather than higher *V*_T_ allows more sensitive detection of changes in the receptor. Because all of our studies on these four radioligands measured *BP*_ND_ using the same method [9–11], here we present a valid comparison of these radioligands.

Data from previous studies demonstrated that, ranked from most to least specific binding, the *BP*_ND_ values (unitless) in HABs were 7.3 for DPA-713, 4.2 for ER176, 1.2 for PBR28, and 0.8 for (*R*)-PK11195 [9–11]. Of note, a *BP*_ND_ value of 0.8 means that specific binding is only 80% of the *V*_ND_, meaning that only 44% (=0.8/(1 + 0.8)) of total uptake (*V*_T_) is specifically bound. Taken together, the results of these human studies clearly show that specific binding of the three second-generation radioligands was much greater than that of the prototypical agent ^11^C-(*R*)-PK11195.

## Sensitivity ^11^C-(*R*)-PK11195 binding to genotype

In PET imaging, all second-generation radioligands, including the three highlighted in this review, are sensitive to varying degrees to the rs6971 SNP. ^11^C-PBR28, DPA-713, and ER176 showed HABs/MABs ratios for *BP*_ND_ of 2.4, 2.1, and 1.2, respectively, whereas (*R*)-PK11195 showed similar *BP*_ND_ for HABs and MABs (Table 1). However, the genotype sensitivity of ^11^C-(*R*)-PK11195 is controversial, and the quantitative measurement of specific binding in human brain provides a potential explanation. Specifically, all reports to date show no statistically significant difference in *V*_T_ of ^11^C-(*R*)-PK11195 among three genotypes (HABs vs MABs vs LABs), but the sample sizes have been small. As we now know, the amount of specific binding in brain of ^11^C-(*R*)-PK11195 is quite small, *BP*_ND_ = 0.8 [10], leading to greater variability and the need for much larger sample sizes to detect statistically significant differences. This theory is supported by the finding that ^11^C-(*R*)-PK11195 is sensitive to genotype in peripheral organs such as lung and heart, which have much greater specific binding than brain [5]. Although direct measurements from brain are not definitive, we suspect that ^11^C-(*R*)-PK11195 is actually sensitive to genotype in brain under in vivo conditions, similar to the pattern observed in peripheral organs, but the low amount of specific binding of this radioligand requires much larger samples sizes to confirm a statistically significant effect. In vitro binding assay using ^3^H-PK11195 showed little sensitivity to the genotype [2]. It should be noted that the sensitivity to the genotype can differ between in vivo (PET) and in vitro binding as we reported for ER176 [3, 11]. A possible cause of the difference is disruption of protein-protein interactions by tissue homogenization for in vitro binding assays.

**Table 1:** Distribution volume and the time stability of four ^11^C–radioligands to image translocator protein (TSPO)

	*V*_T_	*BP*_ND_			Time stability^a^ of *V*_T_	
Ligand	HABs	HABs	MABs	LABs	LABs	HABs after blockade
^11^C-(*R)*-PK11195	0.7	0.8	0.9	0.5	26%	27%
^11^C–PBR28	4.3	1.2	0.5^b^	Not reliably measured	Not reliably measured [6]	25%
^11^C–DPA-713	3.6	7.3	3.4^b^	1.8^b^	25%	13%
^11^C–ER176	3.3	4.2	3.4^b^	1.4^b^	~0%	6%

*HABs* high-affinity binders, *MABs* mixed-affinity binders, *LABs* low-affinity binders, *V*_*T*_ total distribution volume (mL ∙ cm^−3)^, *BP*_*ND*_ specific-to-non-displaceable uptake ratio (unitless)

^a^% increase of *V*_T_ in last 40 min of a 90-min scan (i.e., 50–90 min)

^b^Calculated from *V*_T_ in MABs or LABs and *V*_ND_ measured in HABs after blockade with XBD173 (that is, these *BP*_ND_ values were not measured directly by administering XBD173 in MABs and LABs)

## Radiometabolites that enter brain

PET radioligands tend to be quite lipophilic (log *P* > 3). Relatedly, the radiometabolites of lipophilic drugs—like those typically used for PET imaging of brain—tend to be less lipophilic than their parent compounds and, thus, less likely to enter brain. However, if the radiometabolites of a PET radioligand are lipophilic enough to enter the brain, their contribution to the PET signal will preclude accurate quantitation using only parent radioligand as the input function. In addition, the concentration of parent radioligand typically decreases over time, while that of radiometabolites tends to increase. Thus, if radiometabolites enter the brain, they tend to contribute to an increasing percentage of total brain radioactivity over time. For this reason, one typical pattern that occurs for radiometabolites that enter the brain is that the kinetically determined value of brain binding (i.e., *V*_T_) increases with PET scan duration and never reaches a stable value within the relatively short scan times of 1–2 h. Some radioligands may produce radiometabolite(s) that binds to the target receptor. For these radioligands, accurate quantitation requires more than one input function, i.e., parent and radiometabolite(s). Quantitation using only parent as the single input would cause greater errors at later time points because of increase in radiometabolites.

**Fig. 1:**
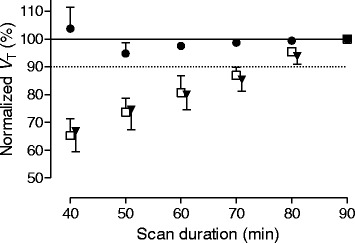
Time-stability analysis. Total distribution volume (*V*_T_) of ^11^C-(*R)*-PK11195 (□), DPA-713 (▼), and ER176 (●) obtained from low affinity binders (LABs) plotted as a function of duration of image acquisition. *V*_T_ from each scan duration is normalized as percentage of *V*_T_ over 90 min of imaging

The effect of radiometabolites accumulating in brain can be observed with the greatest sensitivity when they represent a high percentage of total radioactivity in brain—that is, when parent radioligand represents a small percentage of brain radioactivity. Recent studies examining the four TSPO radioligands of interest presented two scenarios when radiometabolites were a relatively high percentage of brain radioactivity: (1) after administration of XBD173, which blocked uptake of parent radioligand; and (2) in LABs, where low uptake of parent radioligand was caused by the low affinity of TSPO in this genotype. In both of these conditions, we found that *V*_T_ increased with scan duration for three of the four ligands, but not for ER176 (Table 1 and Fig. 1). Combined with a moderate *BP*_ND_ of 1.4 in LABs, an important implication is that LABs do not need to be excluded from studies using ER176. It should be noted here that ER176 is still sensitive to genotype, and that *V*_T_ values must be corrected post hoc; however, LABs need not be excluded a priori.

Notably, radiometabolites are particularly problematic only for LABs, who have a small percentage of parent radioligand in brain. Radiometabolites are much less problematic for HABs and MABs. For example, the specific binding of PBR28—and particularly of DPA-713—is so high (Table 1) that radiometabolites do not preclude their accurate quantitation in HABs and MABs.

Time stability of *V*_T_, i.e., stable *V*_T_ values with longer length of data, is an indirect method to assess accumulation of radiometabolites in brain. A direct method is sampling brain and performing ex vivo analysis in animals. However, even between human and non-human primate, the concentration of radiometabolites is usually markedly different, and TSPO density in brain is also about 20 times different [4, 6] although the structure of TSPO is expected to be similar across species because of high homology of the TSPO gene. Therefore, ex vivo experiments in animals including non-human primate are unlikely to provide good guidance to interpret human data. As a caveat, it should be noted that good time stability of *V*_T_ does not necessarily mean that radiometabolites are not present in brain. For example, if radiometabolites remain a constant percentage of brain radioactivity over time, *V*_T_ will be stable, though it will still be contaminated by these radiometabolites.

## Should low affinity binders be excluded from PET TSPO imaging?

While subjects should not be exposed to radioactivity if their results cannot be used, in our experience, LABs are rare and comprise only 5% of about 500 subjects screened to date at NIH (unpublished data). Nevertheless, excluding LABs is important when studying radioligands like PBR28 because brain uptake is so low that it cannot be quantified. Furthermore, excluding LABs requires a genetic test, which often requires an additional visit to the imaging center or sending a blood sample to a laboratory. Depending on several logistical factors, including distance to the imaging center, rarity of the disorder, and ability of the patient to travel, this screening test that requires an additional visit can be quite problematic.

Based on *BP*_ND_ and time stability of *V*_T_, the data reviewed here indicate that of among these four radioligands—^11^C-(*R*)-PK11195, PBR28, DPA-713, and ER176—the only radioligand that allows LABs to be included in studies of psychiatric and neurological disorders is ER176 although sample sizes were small for all radioligands. Several reasons contributed to this finding. First, studies found that the *V*_T_ of LABs was identified almost as well by the unconstrained two-compartment model as the other genotypes [11]. For whole brain, identifiability (SE) was 0.9% in HABs, 3.4% in MABs, and 1.2% in LABs. Second, the *BP*_ND_ value of ^11^C-ER176 in LABs is quite substantial. For comparison, the *BP*_ND_ of ER176 in LABs (1.4) is comparable to that for PBR28 in HABs (1.2) (Table 1). Third, only ER176 shows time stable values of *V*_T_, consistent with it being the only radioligand not contaminated by radiometabolites accumulating in brain. However, the studies reviewed here had small samples sizes, especially for LABs, and need to be confirmed in much larger groups.

## Conclusions

Among the four TSPO PET ligands reviewed here, ^11^C-DPA-713 had the highest ‘signal to noise ratio’ (*BP*_ND_), but did not provide time stable values of *V*_T_ in LABs, suggesting the accumulation of radiometabolites in brain. ER176 had the second highest *BP*_ND_ value and, importantly, provided time stable values of *V*_T_ in both LABs and HABs after receptor blockade by XBD173. Thus, ER176 has the unique advantage among these four PET radioligands of not needing to exclude LABs a priori, although binding measurements must still be corrected post-hoc for genotype.

## Abbreviations

*BP*_ND_: Specific-to-non-displaceable ratio of distribution volumes
HAB: High-affinity binder
LAB: Low-affinity binder
MAB: Mixed-affinity binder
TSPO: Translocator protein 18 kDa
*V*_ND_: Non-displaceable distribution volume
V_S_: Specific distribution volume
*V*_T_: Total distribution volume
